# Jack of all trades? The versatility of RNA in DNA double-strand break repair

**DOI:** 10.1042/EBC20200008

**Published:** 2020-07-03

**Authors:** Ruth F. Ketley, Monika Gullerova

**Affiliations:** Sir William Dunn School of Pathology, South Parks Road, Oxford OX1 3RE, United Kingdom

**Keywords:** DNA damage, lncRNA, miRNA, RNA modifications, RNA

## Abstract

The mechanisms by which RNA acts in the DNA damage response (DDR), specifically in the repair of DNA double-strand breaks (DSBs), are emerging as multifaceted and complex. Different RNA species, including but not limited to; microRNA (miRNA), long non-coding RNA (lncRNA), RNA:DNA hybrid structures, the recently identified damage-induced lncRNA (dilncRNA), damage-responsive transcripts (DARTs), and DNA damage-dependent small RNAs (DDRNAs), have been shown to play integral roles in the DSB response. The diverse properties of these RNAs, such as sequence, structure, and binding partners, enable them to fulfil a variety of functions in different cellular contexts. Additionally, RNA can be modified post-transcriptionally, a process which is regulated in response to cellular stressors such as DNA damage. Many of these mechanisms are not yet understood and the literature contradictory, reflecting the complexity and expansive nature of the roles of RNA in the DDR. However, it is clear that RNA is pivotal in ensuring the maintenance of genome integrity. In this review, we will discuss and summarise recent evidence which highlights the roles of these various RNAs in preserving genomic integrity, with a particular focus on the emerging role of RNA in the DSB repair response.

## The DNA damage response and double-strand break repair

Our genome is constantly exposed to both exogenous and endogenous genomic threats, such as ionising radiation (IR), ultraviolet radiation (UV), X-rays, reactive oxygen species (ROS) and stalled replication forks, which can lead to a variety of different DNA lesions. These lesions include DNA cross-links, adducts, mismatches, and strand breaks. DNA damage can occur at any point in the genome, and can have detrimental effects on genomic integrity, if unrepaired [1]. In particular, double-strand breaks (DSBs) are considered to be one of the most harmful forms of DNA damage, impairing processes such as replication and transcription, potentially leading to chromosomal translocations, mutations, and cell death. In order to repair genomic insults such as DSBs, the cell employs a complex recognition, signalling, and repair network known as the DNA damage response (DDR). The cell possesses two key pathways for DSB repair, homologous recombination (HR) and non-homologous end joining (NHEJ) [2–5].

**Figure 1:**
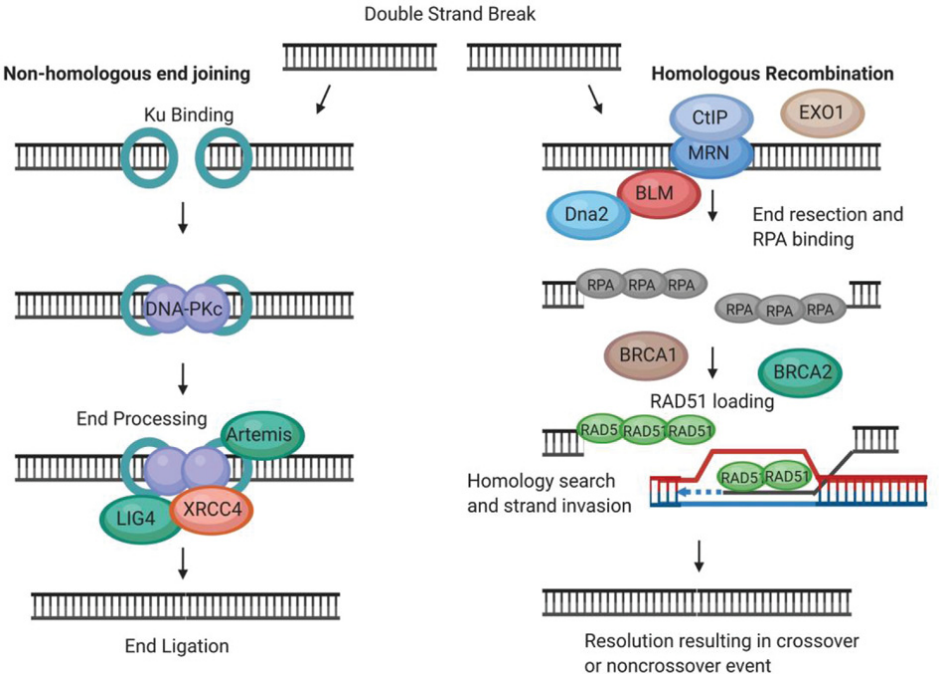
NHEJ and HR are two key pathways for the repair of DSBs The HR pathway of repair involves initial extensive end resection and end processing, RPA binding followed by RAD51 loading, search for a homologous sequence, strand invasion, and resolution. Other proteins involved in HR repair include: the MRN complex, CtIP, BRCA1 and 2, Exo1, Dna2, and BLM. In contrast, in the NHEJ pathway of DSB repair, end resection is prevented, and the break ends require very minimal processing. DNA-PKc, Ku, Lig4, and XRCC4 are some of the proteins known to participate in NHEJ [8]. Image created using Biorender.

HR is an active repair pathway in the late S-phase and G_2_ phase of the cell cycle, when a sister chromatid repair template with sufficient homology is present. HR requires extensive 5′→3′ end resection generating 3′ single-stranded DNA overhangs. The MRN (MRE11–RAD50–NBS1) complex, CtIP, an interacting partner and licensing factor, and BRCA1 promote end resection. MRE11 nicks in close proximity to the DSB and resects in the 3′→5′ direction. 5′→3′ exonucleases, EXO1 and Dna2, are recruited by the MRN complex and digest away from the break. Dna2 resection activity requires BLM to unwind the DNA duplex to provide a resection substrate, and replication protein A (RPA) binds the 3′ overhang ssDNA generated. BRCA2 facilitates RAD51 displacement of RPA and the loading of RAD51 on to the ssDNA. The RAD51-ssDNA nucleofilament then searches for homology and invades the duplex, resulting in the creation of a D-loop structure which can be resolved in a crossover or non-crossover event [2–8](Figure 1).

The critical difference between HR and NHEJ pathway choice is the requirement of end resection for HR, and the prevention of end resection for NHEJ, with end processing being very minimal in the NHEJ pathway in contrast to HR. Antagonism between 53BP1 (anti-resection) and BRCA1 (pro-resection), coupled with the cell cycle phase and other factors such as chromatin context and transcriptional status, helps dictate through which pathway the break is repaired [3,9]. Unlike HR, a homologous template is not a requirement for NHEJ, and NHEJ is active throughout the cell cycle [3]. Initially, Ku binds to the ends of the break, recruiting DNA-PKcs. Subsequently, nucleases and polymerases process the ends for ligation. Artemis is a 5′→3′ exonuclease and forms a complex with DNA-PK, and polymerases μ and λ act in end processing through template-independent nucleotide addition. Lig4 and XRCC4 can ligate together DNA ends of the DSB in complex with Ku, either by blunt-end ligation or following end processing [2–8,10] (Figure 1).

## RNA in DSB repair

It is emerging that RNA has many functions in the repair of DSBs [11–14]. RNA can be either coding messenger RNA (mRNA), translated into proteins, or non-coding RNA (ncRNA), not translated into protein. These ncRNA can be further divided into the classes long non-coding RNA (lncRNA, >200 bp), or small ncRNA (<200 bp) [15]. Small ncRNAs include, but are not limited to, microRNA (miRNA), and the recently identified DNA damage-dependent small RNA (DDRNA) [16]. While RNA processing mechanisms such as RNA splicing and RNA import and export from the nucleus are known to be regulated in response to DSB induction (reviewed in [17,18]); in this review we will be focussing on RNA molecules themselves as regulatory components of the DDR. In particular, we will be discussing the role of both short (miRNA and DDRNA) and long ncRNAs, RNA:DNA hybrid structures, and RNA modifications in mammalian DSB repair.

## MiRNA roles in DSB repair

MiRNAs are ∼22 nucleotide small ncRNAs which have partial sequence complementarity to specific mRNAs. Ribonucleases Dicer and Drosha are responsible for miRNA processing. miRNAs are transcribed as primary transcripts (pri-miRNA) and processed firstly by Drosha and DGCR8 in the nucleus into pre-miRNA. The pre-miRNAs are then exported via Exportin-5 and Ran-GTP into the cytoplasm, where Dicer and TRBP cleave pre-miRNA into mature miRNA, which then are loaded on to Ago proteins to form the RNA-induced silencing complex (RISC) complex. MiRNAs can down-regulate the expression of mRNAs in a process known as RNA interference (RNAi) by guiding RISC to an mRNA with sequence complementarity. mRNA degradation machinery can then be recruited which either disrupts the translational machinery or degrades the target mRNA [11,19].

MiRNA processing is known to be altered in response to DSB induction. For example, Dicer and Drosha binding partners can be regulated by ATM [20–23], ATM can regulate miRNA transcription by phosphorylating transcription factors, and regulate miRNA maturation, processing, and export from the nucleus [24]. Furthermore, DGCR8 can be phosphorylated by c-Abl kinase upon DSB induction [25,26], BRCA1 can associate with Drosha [27] and bind to pri-miRNAs [20,28], and DSBs can induce p53-mediated miRNA transcriptional changes, resulting in differential expression of miRNAs [28]. Over 60% of protein-coding mRNAs are predicted to be targeted by miRNAs [20], and one miRNA can target many different genes [29]. Of these miRNAs, many are now known to play key roles in DSB repair, regulating the levels of a variety of DSB repair factors to augment the DDR to DSBs (Table 1) [11]. Examples of miRNAs regulating DSB repair factors include miR-421, miR-100, and miR-181 regulation of ATM [20,28], miR-24 and miR-138 regulation of H2AX [28,30], and miR-34 family regulation of RAD51 [31]. Furthermore, miRNAs miR-1255b, miR-148b*, and miR-193b* protect genome integrity by promoting DSB repair via the NHEJ pathway in G_1_, through suppression of HR factors BRCA1, BRCA2, and RAD51, preventing loss of heterozygosity (LOH) [32].

**Table 1:** DSB repair factors and miRNAs which target them

DSB repair protein	miRNA	References
H2AX	miR-138 miR-24	[30,33]
ATM	miR-421 miR-101 miR-100 miR-181	[20,28,34–36]
CtIP	miR-130b miR-335	[37,38]
MRE11	miR-493-5p	[39]
MDC1	miR-22	[40]
MAD2L2	miR-890	[29]
53BP1	miR-34a	[41]
BRCA1	miR-1255b miR-148b* miR-193b* miR-182	[32,42]
BRCA2	miR-19a miR-19b miR-1255b miR-148b* miR-193b* miR-19a-3p	[32,43,44]
RAD51	miR-1255b miR-148b* miR-193b* miR-34 miR-96-5p	[31,32,44]
RAD51c	miR-222	[45]
DNA-PKc	miR-101 miR-874-3p	[36,44,46]
Ku80	miR-526b miR-622 miR-218-5p	[44,47,48]
Ku70	miR-502 miR-545 miR-622	[48–50]
Lig4	miR-1246 miR-4727-5p	[51,52]
XLF	miR-502	[49]
GSK3B	miR-21	[53]

Table adapted from [11].

## MiRNA-independent functions of Dicer and Drosha in DSB repair

The canonical miRNA processing functions of Dicer and Drosha have long been established, and their role in miRNA-dependent regulation of DDR factors and DSB factors has been widely studied. However, it is becoming apparent that Dicer and Drosha possess activities in the DSB response which are outside of their canonical miRNA processing roles [19]. While Dicer carries out its miRNA processing in the cytoplasm, a subset of Dicer protein (estimated to be ∼5% of the total dicer pool [54]) has been shown to localise in the nucleus, in order to mediate processing of damage-specific RNA transcripts [55]. Although the nuclear localisation of Dicer has previously been challenged [56], many studies have shown that Dicer can localise in the nucleus [57–59].

In response to DSB induction, Dicer is phosphorylated on residues S1016 and S1728/S1852, and colocalises with γH2AX in the nucleus [55]. Knockdown of Dicer or Drosha reduces foci formation of some DSB repair factors, including 53BP1 and MDC1, in response to IR, suggesting Dicer and Drosha activity is required for DSB repair factor recruitment [16,60]. These DDR foci are sensitive to treatment with RNase A, and can be rescued by re-addition of the small RNA fraction extracted from IR-treated cells, but not Dicer mutant cells. Therefore, Dicer specifically processes small RNAs which are required for DDR focus formation. Using endonuclease site-specific systems, such as the I-SceI-induced DSB system, these RNAs were further characterised as 22–23 nt RNAs originating from the site of the DSB. Synthesis of RNA from sequences surrounding the sequence-specific DSB cut sites was able to rescue 53BP1 and MDC1 foci after RNase A treatment, confirming the sequence-specific nature of the Dicer processing products [16,60]. Taken together, the data suggest that Dicer and Drosha process small RNAs, known as DDRNA, which play a role in the DDR to DSBs [16,60]. These DDRNAs have been suggested to originate from lncRNAs, which are transcribed by RNA polymerase II (RNAPII), actively recruited to the DSB site through interaction with the MRN complex [61,62], both towards and away from the break. Interestingly, the ends of the DSB break can act as promoters for RNAPII transcription [62]. Two differing models have been proposed for RNAPII-dependent transcription at DSB sites. In the first model, damage-induced lncRNA (dilncRNA) are transcribed by RNAPII phosphorylated on the C-terminal domain (CTD) at Serine 2 or 5 (S2P or S5P), a mark of active polymerase transcription. The dilncRNA are either processed by Dicer into DDRNA, or hybridise with the DDRNA, localising them to the DSB site [61]. In the second model, RNAPII is phosphorylated by the kinase c-Abl at the CTD tyrosine position 1 (Y1P) [63]. Y1P RNAPII has been shown to colocalise with γH2AX foci at the sites of promoter-associated DSBs, and is enriched at ASiSI endonuclease cut sites. This Y1P RNAPII transcribes lncRNAs at the DSB site. These lncRNAs then form RNA:DNA hybrids at the DSB site, to promote antisense transcription, production of damage-responsive transcripts (DARTs), and dsRNA production. Accordingly, treatment of cells with either RNase H (RNA:DNA hybrid specific) or RNase III (dsRNA specific) results in reduced 53BP1 foci [63]. While the two models differ slightly in mechanistic detail, it is clear that mounting evidence has underscored the role of RNAPII transcription and Dicer-dependent RNA processing at the sites of breaks in DSB repair, and more specifically in the recruitment of repair factors such as 53BP1 to DSBs. How exactly repair is coordinated by RNA at the break is as yet unclear, and interestingly, γH2AX foci were not shown to be dependent on Dicer and Drosha, which has led to the suggestion that Dicer and Drosha may act in parallel with γH2AX to recruit secondary repair factors [16,60]. It has also been suggested that DDRNA may act to promote phase separation of 53BP1 repair foci to facilitate DSB repair [62]. Together these data suggest that repair factor recruitment to DSBs requires site-specific RNA transcribed at the break [16,55,60–63].

Furthermore, Bonath et al. [64] detect short *de novo* transcription-dependent RNAs originating from I-PpoI-induced DSB sites in mammalian cells of predominantly 21–22 nt in length (referred to as diRNA). However these diRNA could only be detected at sites in repetitive ribosomal regions, but not unique genic or intergenic sites. In contrast, RNAPII-dependent dilncRNA synthesis was observed at both repetitive and non-repetitive sites. This suggests that RNA processing at break sites may differ depending on the genomic context of the DSB. The same study also identified different populations of diRNA, a Dicer-dependent and a Dicer-independent population. The authors also find that Drosha is not required for the production of diRNA, potentially suggesting the repair defects observed upon Drosha knockdown may be due to Drosha activity independent of diRNA/DDRNA processing. Therefore, DDRNA/diRNA production and processing may differ depending on where the DSB occurs, and further work will be required to elucidate the contribution of Dicer and Drosha to DSB repair [64].

## LncRNAs: direct and indirect regulators of DSB repair

**Figure 2:**
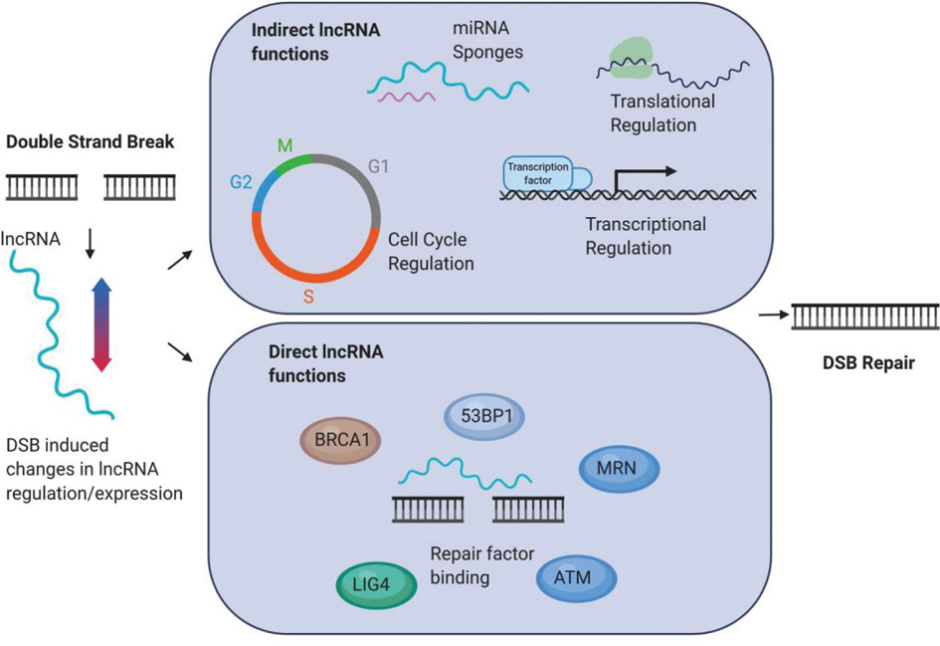
Direct and indirect functions of lncRNAs in response to DNA damage LncRNAs can be differentially expressed or regulated upon DNA damage, and can have a wide range of functions in repair. Examples of lncRNA functions include: at the sites of breaks by recruiting or scaffolding repair factors, in cell cycle regulation, as miRNA sponges, and transcriptional or translational regulation. Image created using Biorender.

There are estimated to be tens of thousands of lncRNAs encoded in the human genome [65,66]. Aside from the lncRNAs dilncRNA and DARTs, a vast array of other lncRNAs have been implicated in the DSB response and the DDR more widely, including in chromatin organisation, cell cycle regulation, and gene expression regulation. The expression of various lncRNAs can be modulated in response to IR [67,68]. LncRNAs also bind DSB repair factors, of which examples include MALAT1, TERRA, and lincRNA-p21 lncRNAs (reviewed in [11]). In this section we will focus on a handful of specific examples of lncRNAs which have recently been implicated in the DDR, both directly, such as through binding to DNA repair proteins, and indirectly, such as by regulating translation of proteins involved in genome stability and DNA repair, highlighting the variety of mechanisms by which lncRNAs can influence repair (Figure 2).

PRLH1 (p53-regulated lncRNA for HR repair 1) is an example of a direct regulator of DSB repair at the site of the break. PRLH1 is p53 regulated and functions in DSB repair pathway choice through its ability to bind RNF169 [69], an E3 ubiquitin ligase, a paralogue of the RNF168. The complex of PRLH1 and RNF169 competes with 53BP1 for binding to RNF168 ubiquitinated chromatin, repressing NHEJ and promoting HR. Knockdown of PRLH1 reduces RNF169 levels, suggesting PRLH1 can regulate HR-mediated DSB repair through stabilisation of RNF169 [69,70]. Consequently, overexpression of PRLH1 lncRNA can promote HR-mediated DSB repair [69,71]. LncRNA HIF-1a inhibitor at translational level (HITT) is another example of a direct DSB regulator. HITT is an ATM interactor, and is up-regulated following DSB induction by EGR1 activity in a p53-independent manner [72]. HITT can augment DSB repair via increased interaction with ATM, reducing the levels of chromatin-bound ATM after DSB induction. Mechanistically, HITT prevents ATM recruitment to DSBs through binding to ATM at the site of NBS1 interaction, blocking ATM and NBS1 association. Consequently, HITT can inhibit DNA end resection through its ability to interact with ATM. HITT has been suggested to be a mechanism of restraining ATM activity in certain cellular contexts, for example after completion of repair, as HITT is up-regulated later relative to ATM activation in the DSB response [72]. PRLH1 and HITT serve as interesting examples of how lncRNAs can be differentially expressed after DSB induction, and can interact directly with key repair proteins such as RNF169 and ATM to regulate DSB repair.

LncRNAs can also act as indirect regulators of DNA repair. One such example of an indirect DNA repair regulator is ncRNA activated by DNA damage (NORAD) [65,73]. NORAD is an lncRNA implicated in preservation of genomic stability, for example via its PUMILIO regulation. PUMILIO proteins are a family of RNA-binding proteins required for a variety of cellular processes [65,74]. PUMILIO proteins bind to mRNA 3′ UTRS in the cytoplasm, stimulating decapping and subsequently down-regulating their translation [65,74]. NORAD was identified as up-regulated in response to DNA damage by Doxorubicin, dependent on p53 [65,75]. PUMILIO proteins bind to NORAD, and are sequestered, resulting in reduced down-regulation of PUMILIO target mRNAs [74,76]. PUMILIO target RNAs shown to be altered in expression upon NORAD depletion include PARP1, BARD1, and EXO1, and other proteins involved in DNA repair, replication, and the cell cycle [65]. Indeed, knockout of NORAD results in aneuploidy and chromosomal instability, hence NORAD is required to maintain genome integrity [65,77]. Other examples of indirect regulators of DNA repair include lncRNA ANRIL, which is activated by ATM signalling after DNA damage, and influences cell cycle progression [78], and lncRNA PANDA, which is up- regulated in a p53-dependent manner after doxorubicin treatment and regulates apoptosis [79]. LncRNAs can also act indirectly in DNA repair as miRNA sponges, competing with endogenous mRNA targets to regulate gene expression, to preserve genome integrity. For example, GUARDIN can sponge miR-23a which targets TRF2 [80], and lnc-RI can compete with miR-4727-5p to regulate Lig4 [52].

These examples highlight how lncRNAs can have multifaceted roles both directly, via direct interaction with DNA repair factors at the site of DSBs, and indirectly, such as influencing transcription of DNA repair factors, cell cycle progression and apoptosis, or as miRNA sponges in the cellular response to damage induction. These and other examples are summarised in Table 2.

**Table 2:** Examples of lncRNAs and their functions in DNA repair

lncRNA	Function	Reference
GUARDIN	Sequesters miR-23a to stabilise TRF2, also scaffolds BRCA1 and BARD1, stabilising BRCA1	[80]
HOTAIR	Regulates miR-218 to influence radiosensitivity	[81]
LIRR1	Up-regulated upon X-ray IR exposure, LIRR1 overexpression decreases expression of DSB repair factors including Ku70 and Ku80	[82]
MALAT1	Forms a complex with PARP1 and Lig3, which are involved in NHEJ, and is required for recruitment of Lig3 to DSB sites	[83]
TERRA	At deprotected telomeres, TERRA binds SUV39H1 H3K9 histone methyltransferase, increasing H3K9me3 and end-to-end fusions	[84,85]
ANRIL	Activated by ATM signalling in response to DNA damage, and is involved in cell cycle regulation	[78]
HITT	Interacts with ATM and restrains HR-mediated DSB repair	[72]
DINO	Interacts with and stabilises p53 in response to doxorubicin treatment	[86]
lincRNA-p21	Regulation of apoptosis via p53 through interaction with hnRNP-K	[87,88]
PCAT-1	Post-transcriptional regulation of BRCA2	[89,90]
PANDA	Upregulated in response to doxorubicin and regulates apoptosis	[79]
LINP1	Translocates from cytosol to nucleus upon IR exposure and scaffolds Ku80 and DNA-PKc	[91,92]
DDSR1	Interacts with BRCA1 to modulate HR	[93]
NORAD	Sequesters PUMILIO proteins, whose target mRNAs include DNA repair and replication proteins, and cell cycle regulators	[65,73–75]
TODRA	RAD51 regulation	[94]
Lnc-RI	Regulates RAD51 expression by competing with miR-193a-3p, also competes with miR-4727-5p to regulate Lig4 expression	[52,95]
CUPID1 and CUPID2	Regulate DNA end resection	[96]
BGL3	Recruited to DSBs and is required for BRCA1-BARD1 accumulation at DSBs	[97]

## R-loops and RNA:DNA hybrids in DSB repair

R-loops are composed of an RNA:DNA hybrid and a single strand of DNA, forming a three-stranded structure [98,99]. R-loops and RNA:DNA hybrids can be a source of genomic instability, as they can impair the replication and transcription machinery [99], and the single-stranded DNA component of the R-loop can be vulnerable to DNA damage [100]. However, RNA:DNA hybrids and R-loops play important roles in class-switch recombination, transcription, and DNA repair, including DSB repair (reviewed in [18,99,101]). DNA:RNA hybrid induction has been observed at areas surrounding DSBs, using different sequence-specific nucleases and model systems including but not limited to; the I-PpoI system in *Schizosaccharomyces pombe (S. pombe*) [102] and humans [103], a fluorescently labelled catalytically inactive RNaseH1 with laser microirradiation in human cells [104,105], in AsiSI U2OS cells with GFP-RNase H1 by ChIP [63], and using the RNA:DNA hybrid specific antibody for immunoprecipitation [63,106]. RNA:DNA hybrids have been suggested to form as a result of dilncRNA synthesis and are involved in the production of DDRNA (discussed above) [63,107].

Factors which have been suggested to influence the accumulation of RNA:DNA hybrids at DSBs include the location of the break, transcriptional status at the site of the DSB, and the downstream repair pathway. RNA:DNA hybrids have been observed preferentially at DSBs in actively transcribed regions of the genome [105,106]. Using the AsiSI cell line system, Cohen et al. observed that RNA:DNA hybrids accumulate at DSBs preferentially in transcribed regions, and more modestly in untranscribed regions [100]. Similarly, Bader et al. find that high transcriptional activity generally correlates positively with hybrid formation at DSBs [108]. As Bader et al. highlighted, intergenic regions are not necessarily transcriptionally silent, and they find that transcriptional activity, but not intergenic or genic location, determines RNA:DNA hybrid formation [108]. However, RNA:DNA hybrids have been detected at some transcriptionally inactive sites, indicating that RNA:DNA hybrids have the potential to form at DSBs even in transcriptionally silent regions of the genome [103,109]. Overall, the evidence suggests transcriptional status may, at least in part, influence RNA:DNA hybrid formation or stability at DSBs.

The contribution of RNA:DNA hybrids preferentially to either HR or NHEJ-mediated DSB repair is unclear. There is evidence to suggest that RNA:DNA hybrids may differentially contribute HR or NHEJ DSB repair pathways. RNA:DNA hybrids have been shown to be required for the recruitment of HR repair factors to DSBs, including BRCA1, BRCA2 [103], and RAD52 [105], and numerous studies have shown that modulation of RNA:DNA hybrid formation and processing impacts HR-mediated DSB repair [100,102,103,105,110,111], suggesting a more prominent role for RNA:DNA hybrids in HR. However, in some instances RNA:DNA hybrids have been shown to form at sites repaired by both NHEJ and HR [106,108]. Drosha can promote RNA:DNA hybrid accumulation at DSBs, and Drosha depletion attenuates RNA:DNA hybrid formation, impairing both HR and NHEJ repair [106]. The intrinsically disordered protein RBM14 was shown to be required for the formation of RNA:DNA hybrids at DSB sites, and knockdown of RBM14 reduces NHEJ, implicating RNA:DNA hybrids in NHEJ-mediated DSB repair [109].

While a growing body of evidence has underscored the importance of RNA:DNA hybrids in DSB repair, a failure to process RNA:DNA hybrids at DSBs can lead to DNA damage and genome instability. This highlights that while RNA:DNA hybrids play a key role in repair, they must be tightly regulated. For example, Senataxin, an RNA:DNA helicase, is recruited to RNA:DNA hybrids at DSBs in transcriptionally active regions, but not intergenic or transcriptionally silent regions. Senataxin acts to prevent potential translocations at break ends in transcriptionally active loci, possibily by resolving RNA:DNA hybrids [100]. Furthermore, depletion of Senataxin or RNA:DNA hybrid processing enzymes RNase H1 and H2 in *Saccharomyces cerevisiae* (*S. cerevisiae*) results in cell cycle arrest and DNA damage [112]. While transient RNA:DNA hybrids are required to regulate end resection in *S. pombe*, the depletion of RNase H1, and subsequent lack of RNA:DNA hybrid removal, impairs RPA loading to ssDNA at the DSB site [102]. BRCA2 also recruits RNase H2 to regulate RNA:DNA hybrid levels at DSBs [56]. HNRNPD, an RNA-binding protein whose knockdown impairs HR, is required to resolve RNA:DNA hybrids in order to facilitate DNA end resection [111]. Moreover, EXOSC10, an RNA exosome subunit, processes RNA:DNA hybrids which occur at DSBs from dilncRNAs, necessary for end-resection regulation and RPA binding to ssDNA for HR-mediated DSB repair [107,110,113]. This suggests that while RNA:DNA hybrids are an important component of repair, their regulation and timely removal by ribonucleases such as Senataxin, EXOSC10, and RNase H enzymes, is necessary for proper DSB repair.

RNA:DNA hybrids play a key role in DSB repair, although their generation and regulation must be carefully controlled in order to preserve genome integrity. Data suggest that the role of RNA:DNA hybrids may be context specific, potentially depending on chromatin context, transcriptional status, and cell cycle phase [14].

## Looking ahead: RNA modifications and the DDR. A role for RNA modifications in DSB repair?

**Figure 3:**
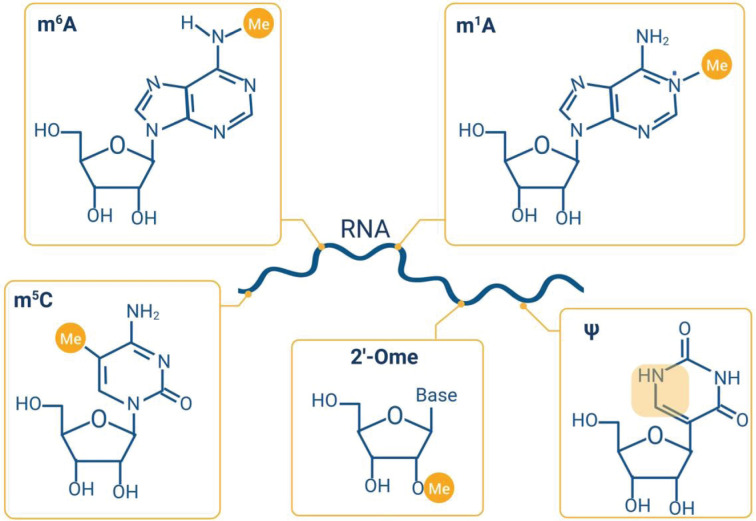
The structures of modifications which can be found in RNA m^6^A, m^1^A, m^5^C, 2′-OMe, and Ψ are depicted. It is now understood that RNA modifications can influence the stability and structure of RNAs, alter protein–RNA interactions, and therefore regulate the functionality of many RNAs. Image created using Biorender.

It is now understood that RNA itself, much like DNA and proteins, can be modified post-transcriptionally. These RNA modifications possess the ability to alter a diverse array of cellular processes, including cell cycle progression and apoptosis, by altering the stability and structure of RNAs, and protein–RNA interactions. Modifications of RNA include; N^6^-methyladenosine (m^6^A), N^1^-methyladenosine (m^1^A), 5-methylcytosine (m^5^C), 2′-O-methylation (2′-OMe), and pseudouridine (Ψ), however over 150 RNA modifications have been identified (Figure 3). Many different RNAs can be modified, such as transfer RNAs (tRNAs), ribosomal RNAs (rRNA), mRNAs, and lncRNAs [114,115]. Recently, RNA modifications have been suggested to play a role in the cellular response to DNA damage [116].

m^6^A, found in both DNA and RNA, is the most abundant internal RNA modification found in cells, and known functions include in mRNA splicing [117,118], translation [119–121], and cell cycle regulation [122,123]. The RNA m^6^A modification is reversible, and has several writers (the METTL3–METTL14 complex, METTL16, WTAP), and erasers (ALKBH1/3/5, and FTO) [118,124–127]. m^6^A has also been implicated in the cellular stress response, DNA repair, and cancer progression. LncRNA LNCAROD, which is up-regulated in HNSCC, is highly m^6^A modified by METTL3 and METTL14 in these samples. m^6^A modification stabilises LNCAROD, and LNCAROD expression correlates with poor prognosis and tumorigenicity in HNSCC [128]. In gastric cancer, m^6^A-associated genes were found to be dysregulated, and poor prognosis correlated with high expression of FTO and WTAP [129]. Moreover, m^6^A can modulate translation in acute myeloid leukemia [130], and in response to heat shock [131]. m^6^A demethylase FTO has also been implicated in the DDR and the stress response in mice osteoblasts, where FTO knockout led to an increase in DNA damage and apoptosis in response to genotoxic stress, suggesting a role for m^6^A and FTO in the cellular stress response [132]. Recent studies have found that m^6^A has a further role in the DDR in the repair of UV-induced DNA damage, specifically in the nucleotide excision repair (NER) pathway. The RNA m^6^A-modification accumulates rapidly at UV laser microirradiated sites, colocalising with the m^6^A writer METTL3–METTL14 complex. More generally, UVC induced m^6^A in a variety of transcripts, indicating a possible dual role of m^6^A in the UV DDR, both at the site of the damage and more globally within the cell [133,134]. m^6^A was found to be required for the recruitment of downstream repair factors, such as Polymerase κ (Pol κ), although DSB repair factors such as BRCA1 and 53BP1 were not dependent on the m^6^A writer METTL3 for recruitment to damage [133]. However, it is now understood that the m^6^A modification is required for the DDR to UV damage, providing proof of principle that RNA modifications can function in the DDR. A role for m^6^A at DSB sites is yet to be established, although m^6^A levels have been found to be altered in response to DSBs [127,135]. m^6^A and METTL3 were found to be elevated in glioma stem-like cells in response to IR, and silencing of METTL3 reduced DSB repair and enhanced the sensitivity to IR in these cells [135]. Moreover, the lncRNA pncRNA-D m^6^A modification levels were found to be reduced in response to IR or osmotic stress, which can induce DSBs, suggesting m^6^A can be modulated in response to DSBs [127].

Furthermore, m^6^A has been shown to be present on RNA:DNA hybrids [136,137]. Abakir et al. have shown that m^6^A can coordinate R-loop removal with the m^6^A reader, YTH-domain family member 2 (YTHDF2), contributing to genome stability [136,138]. Interestingly, this m^6^A modification of R-loops is not constitutive, but rather is cell cycle dependent. m^6^A is present on R-loops in S and G_2_/M phases of the cell cycle, and is reduced in the G_0_/G_1_ phases of the cell cycle [136,138]. It is not yet clear if this could impact the DDR, in particular the DSB response, but given the known role of R-loops in DSB repair [63,100,111], it implicates a further possible role for m^6^A in the cellular DDR.

Another RNA modification with roles in the cellular stress response is m^1^A. m^1^A is present on RNA at approximately 5–10% that of m^6^A, and can be found in tRNA, rRNA, and has recently been identified in mRNA and lncRNA [139–142]. m^1^A on various RNAs can be modulated in response to stress conditions, such as hydrogen peroxide treatment which can induce strand breaks in DNA. Interestingly, this m^1^A RNA modification can be reversed by ALKBH3, an enzyme with a known role in DNA repair [140]. While m^1^A has not been shown to be recruited to UV-microirradiation in the same manner as m^6^A [134] further studies may shed light on a potential role of in the DDR. However, it is important to note that there has been controversy regarding the m^1^A antibody, suggesting m^1^A peaks identified in 5′UTRs are likely the result of antibody cross-reactivity with the m^7^G-cap of RNA [143].

Furthermore, ADP-ribosylation, a modification known to be involved in a variety of cellular processes [144], including the repair of DSBs, has been identified as a reversible RNA modification [145]. ADP ribosylation, where an ADP-ribose group is added to a molecule, was previously only known to be present on DNA and proteins. However, Munnur et al. have identified that RNA can be ADP-ribosylated, and this modification can be removed by a variety of enzymes, including NUDT16 [145], which has recently been shown to play a role in DSB repair [146]. While RNA ADP-ribosylation has only been shown *in vitro*, it is interesting to speculate about its possible role in cells in the DDR, given the importance of ADP-ribosylation of DNA and protein in DSB repair and RNA biology [144,147].

## Conclusion

RNAs play roles in the repair of DSBs at multiple levels. The specific features of each of the RNAs, including sequence, structure, and binding partners, enable RNA to have a wide variety of functions both at the sites of DSBs and more generally in the DDR. These roles include direct binding of repair factors both at the sites DSBs and elsewhere in the cell, post-transcriptional regulation of repair factor expression, and regulation of end resection. Furthermore, understanding how ways of altering RNA properties, such as RNA modification, can impact DNA repair will likely be an area of expanding interest. New technologies to map RNA modifications such as sequencing technologies [148] and mass spectrometry analysis tools [149] will enable the further understanding of the cellular role of RNA modifications. Elucidating the many ways in which RNA can influence DNA repair processes is of importance for understanding how RNAs can influence tumorigenesis and cancer progression, and ultimately could be targeted for cancer therapeutics [150].

## Summary

- DSBs are considered one of the most cytotoxic forms of DNA damage, and their repair is critical to preserve genomic information.
- RNA is emerging as a key player in the DSB repair response.
- MiRNAs, lncRNAs, RNA:DNA hybrids, dilncRNAs, DARTs, and DDRNAs act through various mechanisms, such as in transcriptional gene silencing, as scaffolds for proteins, and signalling molecules, to augment the DSB response and maintain genomic integrity.
- Modification of RNA is emerging as a mechanism of regulation in DNA repair. We speculate RNA modifications are an area of future research in the DSB repair field.

## Open Access

Open access for this article was enabled by the participation of University of Oxford in an all-inclusive *Read & Publish* pilot with Portland Press and the Biochemical Society under a transformative agreement with JISC.

## Abbreviations

2′-OMe: 2′-O-methylation
m^5^C: 5-methylcytosine
CTD: C-terminal domain
DART: damage-responsive transcript
DDR: DNA damage response
DDRNA: DNA damage-dependent small RNA
dilncRNA: damage-induced long non-coding RNA
DSB: double-strand break
HITT: HIF-1a inhibitor at translational level
HR: homologous recombination
IR: ionising radiation
lncRNA: long non-coding RNA
miRNA: microRNA
MRN: MRE11–RAD50–NBS1
mRNA: messenger RNA
m^1^A: N^1^-methyladenosine
m^6^A: N^6^-methyladenosine
ncRNA: non-coding RNA
NHEJ: non-homologous end joining
NORAD: ncRNA activated by DNA damage
pri-miRNA: primary transcript of miRNA
PRLH1: p53-regulated lncRNA for HR repair 1
RISC: RNA-induced silencing complex
RNAPII: RNA polymerase II
RPA: replication protein A
rRNA: ribosomal RNA
tRNA: transfer RNA
UV: ultraviolet radiation
